# Prevalence of Linda Virus Neutralizing Antibodies in the Austrian Pig Population

**DOI:** 10.3390/v13061001

**Published:** 2021-05-27

**Authors:** Alexandra Kiesler, Jakob Plankensteiner, Lukas Schwarz, Christiane Riedel, Kerstin Seitz, Marlene Mötz, Andrea Ladinig, Benjamin Lamp, Till Rümenapf

**Affiliations:** 1 Department for Pathobiology, Institute of Virology, University of Veterinary Medicine, Veterinaerplatz 1, 1210 Vienna, Austria; Alexandra.Kiesler@vetmeduni.ac.at (A.K.); 01316377@students.vetmeduni.ac.at (J.P.); Christiane.Riedel@vetmeduni.ac.at (C.R.); Kerstin.Seitz@vetmeduni.ac.at (K.S.); Marlene.Moetz@vetmeduni.ac.at (M.M.); 2 Department for Farm Animals and Veterinary Public Health, University Clinic for Swine, University of Veterinary Medicine, Veterinaerplatz 1, 1210 Vienna, Austria; Lukas.Schwarz@vetmeduni.ac.at (L.S.); Andrea.Ladinig@vetmeduni.ac.at (A.L.); 3 Institute of Virology, Faculty of Veterinary Medicine, Justus-Liebig-University Giessen, Schubertstrasse 81, 35392 Giessen, Germany

**Keywords:** pestiviruses, Linda virus, seroprevalence, serum virus neutralization assay, novel Linda virus strain

## Abstract

A novel pestivirus species, termed Lateral-shaking Inducing Neuro-Degenerative Agent virus (LindaV), was discovered in a piglet-producing farm in Austria in 2015 related to severe congenital tremor cases. Since the initial outbreak LindaV has not been found anywhere else. In this study, we determined the seroprevalence of LindaV infections in the domestic pig population of Austria. A fluorophore labeled infectious cDNA clone of LindaV (mCherry-LindaV) was generated and used in serum virus neutralization (SVN) assays for the detection of LindaV specific neutralizing antibodies in porcine serum samples. In total, 637 sera from sows and gilts from five federal states of Austria, collected between the years 2015 and 2020, were analyzed. We identified a single serum showing a high neutralizing antibody titer, that originated from a farm (Farm S2) in the proximity of the initially affected farm. The analysis of 57 additional sera from Farm S2 revealed a wider spread of LindaV in this pig herd. Furthermore, a second LindaV strain originating from this farm could be isolated in cell culture and was further characterized at the genetic level. Possible transmission routes and virus reservoir hosts of this emerging porcine virus need to be addressed in future studies.

## 1. Introduction

Pestiviruses are enveloped, small viruses with a positive-sense, single-stranded RNA genome of about 12.3 to 13 kb length [1]. The RNA genome consists of one large open reading frame (ORF) that is flanked by a 5′- and 3′-untranslated region (UTR). The ORF codes for a polyprotein, which is co- and post-translationally processed into four structural proteins, namely Core, E^rns^, E1 and E2, and eight non-structural proteins N^pro^, p7, NS2, NS3, NS4A, NS4B, NS5A and NS5B [1]. Within the family *Flaviviridae*, the envelope glycoprotein E^rns^ and the auto-protease N^pro^ are a unique characteristic for the genus *Pestivirus*, although a recently discovered pestivirus species in toothed whales lacks the N^pro^ gene [2].

The genus *Pestivirus* currently comprises 11 different species—recently termed *Pestivirus A*–*K* [3]. In addition to the classical pestivirus species, bovine viral diarrhea virus 1 (BVDV-1, *Pestivirus A*), bovine viral diarrhea virus 2 (BVDV-2, *Pestivirus B*), classical swine fever virus (CSFV, *Pestivirus C*), and border disease virus (BDV, *Pestivirus D*), several novel pestiviruses have been identified in different host species. Three new species of pestiviruses have been found in domestic pigs in the last two decades, causing various forms of disease. Diverse strains of atypical porcine pestiviruses (APPV, *Pestivirus K*) that induce congenital tremor of type A-II in newborn piglets after intrauterine infection have been identified in shaking piglets worldwide [4,5,6]. A multitude of studies on the prevalence of APPV revealed a wide geographic distribution and an overall high prevalence in the domestic pig population as well as in the wild boar population [7,8,9,10,11]. Currently, APPV is separated into three clades (Clade I-III) [12]. While in North America and Europe Clade I prevails, Clades II and III are abundant in China and neighboring countries. In contrast, the only known strain of the species Bungowannah virus (BungoV, *Pestivirus F*) appeared in Australia and caused the so-called porcine myocarditis syndrome [13]. BungoV became endemic in one of the affected farm complexes [14], but it has never been found anywhere else [8,10,15,16]. A further porcine pestivirus was identified in 2015 in Austria during a screening for APPV in samples of piglets with congenital tremor. The clinical symptoms of Linda virus are reminiscent of congenital tremor in newborn piglets, but the affected piglets showed a stronger shaking phenotype with a higher pre-weaning mortality rate and the identified pestivirus was therefore termed Lateral-Shaking Inducing Neuro-Degenerative Agent (Linda) virus (LindaV, tentatively *Pestivirus L*) [17]. Phylogenetically, BungoV and LindaV are more closely related to each other than to any other pestivirus. Interestingly, the newly discovered whale pestivirus Phocoena pestivirus belongs to the same branch as LindaV and BungoV [2]. As observed with BungoV, LindaV has never been detected again since its first description.

So far, epidemiological studies regarding the prevalence of LindaV in the pig population have focused on the detection of LindaV RNA in porcine serum samples [9,10]. Our recent results from animal studies demonstrated that acute infections with LindaV are difficult to detect in immunocompetent animals despite the persistence of the virus in the tonsils and lymphoid organs [18]. These findings are similar to acute BVDV infections, where direct virus detection by RT-PCR is a diagnostic challenge, as only very low viral loads are detectable in serum samples [19]. Therefore, the absence of LindaV RNA in serum samples is not likely to be sufficiently sensitive to conclude an absence of the virus in a population. An experimental infection of immunocompetent pigs with LindaV induced a strong humoral immune response with high neutralizing antibody titers, which presumably last for a longer period of time. Cross-neutralization of antibodies with other pestivirus species was not observed except for BungoV specific antibodies [18]. Therefore, epidemiological studies based on the detection of LindaV neutralizing antibodies represent a reliable tool to gain insights into the prevalence of LindaV infections in the pig population.

Serum virus neutralization (SVN) assays are seen as the gold standard in serological diagnostics of pestiviral infections with regard to specificity and sensitivity of antibody detection. The combination of different pestiviral species and strains in comparative SVN assays for the detection of potential cross-neutralization and high-titer specific neutralization allows a precise indirect virus diagnosis [20,21,22]. Unfortunately, SVN assays represent a laborious and time-consuming diagnostic test that limits the mass screening of serum samples. To overcome these limitations, a fluorophore encoding LindaV clone (mCherry-LindaV) was constructed based on an infectious cDNA clone of LindaV, which allows a direct readout of the assay without the need for immunofluorescence staining.

In this study, we assessed the seroprevalence of LindaV infections in the domestic pig population of Austria. Porcine serum samples from commercial pig farms were screened for the presence of LindaV specific neutralizing antibodies in an SVN assay using an mCherry-LindaV clone for rapid analysis. Additionally, the serum samples were analyzed in a LindaV specific RT-qPCR. The introduction of LindaV in naïve pig herds is a considerable threat, potentially leading to major piglet losses, as was seen on the originally affected farm. Therefore, knowledge of the presence of this virus in the pig population is of high importance for pig producers.

## 2. Materials and Methods

### 2.1. Cells

SK-6 cells [23] were grown in Dulbecco’s modified Eagle’s medium (DMEM, Biowest, Nuaillé, France) supplemented with 10% heat-inactivated fetal calf serum (FCS, Corning, Tewksbury, MA, USA; negatively tested for pestiviruses), 100 U/mL penicillin and 100 µg/mL streptomycin. Cells were maintained at 37 °C and a CO_2_ concentration of 5%.

### 2.2. Indirect Immunofluorescence Assays

Indirect immunofluorescence assays were performed as previously described [17]. Briefly, the cells were fixed with 4% paraformaldehyde for 20 min at 4 °C, permeabilized with 1% (vol/vol) Triton-X 100 (Merck, Darmstadt, Germany) in PBS, and stained with the cross-reactive mouse monoclonal antibody (MAb) 6A5 (anti E2). Goat anti-mouse IgG conjugated with Cy3 (Dianova, Hamburg, Germany) or goat anti-mouse IgG conjugated with FITC (Dianova) were used as secondary antibodies. Cell nuclei were counterstained with Hoechst 33342 (Thermo Fisher Scientific, Waltham, MA, USA) at a concentration of 5 µg/mL for 5 min at room temperature.

### 2.3. Generation of a Full-Length Linda Virus cDNA Clone

The initial LindaV field isolate from a concentrated passage three master stock (5 × 10^8^ TCID_50_/mL) was chosen for our molecular cloning attempts to avoid cell culture adaptations within the genome of the virus. A total of 96 mL of a LindaV suspension (passage 4) was concentrated using ultracentrifugation applying an average centrifugal field of 95,800× *g* for 4 h at 4 °C (Beckman Type 45 Ti rotor, 35,000 rpm). The pellet was resuspended in 400 µL Hepes buffer (25 mM, pH 7.5) and the total RNA was purified using the RNeasy Mini Kit (QIAGEN, Hilden, Germany). The LindaV genome sequence (GenBank accession number: KY436034.1) was presented earlier [17], allowing the design of oligonucleotides hybridizing with the 5′-end and the 3′-end of the genome. A full-genomic cDNA fragment was amplified by RT-PCR using oligonucleotides LindaV-5′-forw (5′-GTATAGCAGCAGTAGCTCAAGGCTG-3′) and LindaV-3′-rev (5′-GGGCCTCTTGGAACTGTAAGTAGTC-3′) and the One*Taq* One-Step RT-PCR Kit (NEB, Ipswich, MA, USA). A pBR322 derived vector already containing all the features necessary for RNA translation including an SP6 promoter and a XhoI site for linearization was amplified by extension PCR to provide homologous sequence patches for cloning of the cDNA. Q5 polymerase (NEB) was used for vector amplification together with the oligonucleotides LindaV-VGA-forw (5′-CAGTTCCAAGAGGCCCCTCGAGCTACCTCACTAACG-3′) and LindaV-VGA-rev (5′-GCTACTGCTGCTATACTATAGTGTCACCTAAATCGC-3′). The PCR products of 12.6 kb (viral cDNA) and 2.1 kb (vector) were purified (Monarch DNA gel extraction kit, NEB) and combined to generate plasmid pL588 using a DNA assembly reaction (NEBuilder, NEB). For differentiation of the cloned recombinant LindaV (recLindaV) and the LindaV field isolate, a novel MluI site at nt position 5587 was introduced. Extension PCR was performed with Q5 polymerase (NEB) and oligonucleotides LindaV-MluI-forw (5′-AAAACGCGTGGCGCTATGGTACACCTCAGAAAAACAGGTC-3′) and LindaV-MluI-rev (5′-TTTACGCGTGCCTGCTATGTTGACGGCTTCGGGATTTATTAC-3′), which preserved the encoded amino acid sequence. The PCR product was digested with MluI, purified and ligated with T4 ligase (NEB) resulting in plasmid pL602. With the help of the primers LindaV-4868-forw (5′-CAGCAGACAGCAACAGTATAC-3′) and LindaV-5901-rev (5′-CTTCCCTGCCCCAGTTGCTAG-3′), a 1055 nt fragment was amplified flanking the MluI site. The RT-PCR products were diluted 1:10 in 1 x restriction enzyme buffer (NEB3.1, NEB), digested with MluI at 37 °C for 1 h and subjected to gel electrophoresis.

The viral cDNA clone was passaged in the *E. coli* strain HB101, DNA was prepared by standard methods and the genome of recLindaV was confirmed by sequencing.

### 2.4. RNA In Vitro Synthesis and Virus Rescue

Synthetic infectious RNA was produced as previously described [24]. Briefly, 2.5 µg DNA of the plasmids pL588 and pL602 were digested with XhoI and purified using phenol-chloroform extraction. The linearized plasmid DNA was transcribed into genomic recLindaV RNA using SP6 polymerase (NEB). A total volume of 50 µL of the transcription mixture was DNase digested. The RNA was purified with the RNeasy Mini Kit (QIAGEN), eluted in RNase free water, and diluted with water to a final concentration of 0.25 µg/µL. SK-6 cells were transfected with 2.5 µg of the synthetic RNA by electroporation as previously described [25] and incubated for 24 h until progeny virus was harvested from the supernatant.

### 2.5. Construction of a Fluorophore Labeled Full Genome Infectious cDNA Clone of Linda Virus

**Figure 1:**
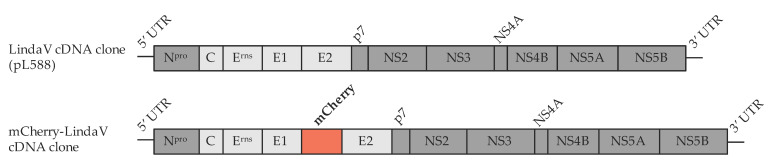
Schematic representation of the construction of an mCherry-labeled full genome infectious cDNA clone of Linda virus (LindaV). The coding sequence of the fluorescent protein mCherry was fused to the 5′-end of the LindaV E2 coding sequence in a LindaV cDNA backbone using a DNA assembly reaction. Non-structural protein coding sequences are shown in dark grey, and structural protein coding sequences in light grey. Lines represent 5′- and 3′-untranslated regions of the genome. UTR, untranslated region.

Based on the full genome infectious cDNA clone of LindaV (pL588, as described in Section 2.3), a fluorophore labeled infectious cDNA clone of LindaV was constructed by fusing the coding sequence of the fluorescent protein mCherry to the 5′-end of the LindaV E2 coding sequence behind the signal peptide (Figure 1). PCR products with overlapping ends harboring the mCherry sequence were generated with the primers mCherry-F (5′-TAATAGGGGGAGCCCAGGGTATGGTGAGCAAGGGCGAGGAG-3′) and mCherry-R (5′-GCAGTTCGAAGTTGCATTCAAGCTTGTACAGCTCGTCCAT-3′). The LindaV cDNA backbone was amplified with the primers LindaV-E2-F (5′-ATGGACGAGCTGTACAAGCTTGAATGCAACTTCGAACTGC-3′) and LindaV-E1-R (5′-CTCCTCGCCCTTGCTCACCATACCCTGGGCTCCCCCTATTA-3′). PCRs were performed using Q5 DNA Polymerase (NEB). The PCR fragments were assembled in a DNA assembly reaction (NEBuilder; NEB) according to the manufacturer’s instructions.

### 2.6. Serum Samples

The sample size for our sero-epidemiological study was determined based on the following Formula (1):

n ≤ [1 − (1 − ε)^1/d^] × [N − (d − 1)/2] (1)

where n = sample size number; ε = confidence level, set to 95%; d = number of diseased animals in the population; and N = population size [26]. Two different calculations with an assumed prevalence of 0.5% were made, one based on the total number of breeding animals in Austria (234,000 animals) and the other based on the total number of domestic pigs kept in Austria (2,770,000 animals). Both calculations resulted in an almost identical number of 597 and 598, respectively, porcine sera to be screened.

In total, 637 porcine serum samples from 132 pig farms, provided by the University Clinic for Swine of the University of Veterinary Medicine, Vienna and the Veterinary Health Service in Upper Austria, were screened for the presence of LindaV neutralizing antibodies and LindaV RNA. The samples originated from sows and gilts from pig farms located in five federal states of Austria, namely Upper Austria (n = 335), Styria (n = 214), Lower Austria (n = 67), Carinthia (n = 11) and Burgenland (n = 10). The sera were taken by farm veterinarians during routine herd health monitoring visits between the years 2015 and 2020. Additional serum samples (n = 57) from the LindaV positive farm in Styria, identified during this study, were obtained and analyzed. Sera from the year 2016 (n = 30; 20 post-weaning piglets and 10 fattening pigs) were collected within the frame of a Porcine Reproductive and Respiratory Syndrome Virus (PRRSV) circulation study, whereas sera from 2019 (n = 7; five post-weaning piglets, one gilt and one sow) and 2021 (n = 20, 10 fattening pigs and 10 of unknown origin) were sent in for diagnostic purposes. All serum samples were stored at −20 °C. Sera were heat inactivated for 30 min at 56 °C prior to conducting the SVN assays.

### 2.7. Serum Virus Neutralization (SVN) Assay

Initially, serum dilutions of 1/5 and 1/10 were prepared in DMEM without FCS in 96-well cell culture plates (STARLAB, Hamburg, Germany) in duplicate. An mCherry-LindaV stock (1.78 × 10^5^ TCID_50_/mL, determined by end-point dilution assay) was diluted to a titer of 100 TCID_50_/50 µL. The test virus was added to the serum dilutions and incubated at 37 °C for 2 h. 1 × 10^4^ SK-6 cells were seeded directly into the wells containing the pre-incubated serum/virus-mixture and grown for 72–96 h post infection. Defined positive and negative reference antisera, obtained from an experimental infection of immunocompetent pigs with LindaV [18], serum toxicity controls (serum dilution 1/5), cell controls and virus back titration controls were included in each SVN assay. Cells were fixed with 4% paraformaldehyde in PBS for 20 min at 4 °C, when a strong fluorescence signal was detectable in wells containing the negative reference sera and directly analyzed using a fluorescence microscope (Olympus IX70 fluorescence microscope; OLYMPUS, Hamburg, Germany).

Sera showing neutralizing activity in both initial dilutions were analyzed again in a five-fold serial dilution starting at a dilution of 1/5 and reaching a final dilution of 1/390,625. The 50% neutralization dose (ND_50_/mL) was calculated using the Spearman-Kaerber method and expressed as the reciprocal (1/ND_50_/mL) of the serum dilution.

### 2.8. RT-qPCR

Serum samples were pooled to a total volume of 140 µL (5 sera/pool; 28 µL per serum). Total RNA was extracted using the QIAamp Viral RNA Mini Kit (QIAGEN) according to the manufacturer’s instructions. RT-qPCRs were performed on a Rotor-Gene Q cycler (QIAGEN) using the Luna Universal Probe One-Step RT-qPCR Kit (NEB). LindaV as well as BungoV specific primers and probe were used as previously described [18]. Sera of positive pools were extracted separately using 140 µL of each serum and again analyzed in RT-qPCR. The housekeeping gene beta-actin was used as an internal control for proof of successful RNA extraction and the absence of inhibitory factors in RT-qPCR. Amplification of beta-actin was conducted in a separate RT-qPCR run using the primers beta-actin-F1 (5′-CAGCACAATGAAGATCAAGATCATC-3′), beta-actin-R2 (5′-CGGACTCATCGTACTCCTGCTT-3′) and the probe beta-actin-HEX (5′-HEX-TCGCTGTCCACCTTCCAGCAGATGT-BHQ-1-3′) under the same cycling conditions used in the LindaV RT-qPCR.

### 2.9. Two-Step RT-PCR and Sanger Sequencing

In order to obtain a full sequence of serum samples in which LindaV RNA could be detected, a set of primer pairs covering the full genome of LindaV was designed based on the available LindaV sequence in GenBank (accession number KY436034.1, oligonucleotides presented in Table S1). At first, cDNA was synthesized from 5 µL RNA using the HiScript II 1st Strand cDNA Synthesis Kit (Vazyme Biotech, Nanjing, China) according to the manufacturer’s instructions. The cDNA was purified using Quantum Prep PCR Kleen Spin Columns (Bio-Rad, Hercules, CA, USA) and 2.5 µL cDNA served as a template in subsequent PCRs using Q5 DNA Polymerase (NEB). PCR products were purified using the Monarch PCR DNA Cleanup Kit (NEB). Sanger sequencing of purified amplicons was performed by Eurofins Genomics, and sequence analysis was done using the DNA Strider 3.0 software [27,28].

### 2.10. Virus Isolation

A volume of 100 µL serum was used to inoculate 5 × 10^4^ SK-6 cells, grown in DMEM with 10% FCS and penicillin/streptomycin on a 24-well cell culture plate. Cells were incubated at 37 °C and passaged every 72 h and cell culture supernatant was used to infect fresh cells in parallel. After every passaging, cells were examined for the presence of viral antigen in indirect immunofluorescence assays (as described in Section 2.2) using the cross-reactive mouse MAb 6A5 (anti E2) and goat anti-mouse IgG conjugated with Cy3 (Dianova) as a secondary antibody.

Additionally, total RNA was extracted from 140 µL cell culture supernatant using the QIAamp Viral RNA Mini Kit (QIAGEN) and successful virus propagation was identified by decreasing Ct values in the LindaV RT-qPCR.

### 2.11. Phylogenetic Analysis

Phylogenetic analysis of the novel LindaV strain (GenBank accession number: MZ027894) was performed using CLC Sequence Viewer 7.7.1 (CLC bio/QIAGEN Digital Insights, Aarhus, Denmark) based on the full-genomic nucleotide sequence or the polyprotein sequence. Sequences of approved and unclassified pestivirus species available in GenBank were used for sequence comparison. GenBank accession numbers of the respective pestivirus species are as follows: Linda virus (KY436034.1, tentatively *Pestivirus L*), Bungowannah virus (EF100713.2, *Pestivirus F*), CSFV Alfort_187 (X87939.1, *Pestivirus C*), BVDV-1 NADL (M31182.1, *Pestivirus A*), BVDV-2 890 (U18059.1, *Pestivirus B*), BDV X818 (AF037405.1, *Pestivirus D*), sheep pestivirus Aydin (NC_018713.1, *Pestivirus I*), pronghorn antelope pestivirus (NC_024018.2, *Pestivirus E*), reindeer pestivirus (AF144618.2, *Pestivirus D*), giraffe pestivirus (NC_003678.1, *Pestivirus G*), BVDV-3 D32_00_HoBi (AB871953.1, *Pestivirus H*), APPV AUT-2016_C (KX778724.1, *Pestivirus K*), *Rhinolophus Affinis* pestivirus 1 (JQ814854.1, unclassified), Norway rat pestivirus (KJ950914.1, *Pestivirus J*) and Phocoena pestivirus isolate NS170386 (MK910229.1, unclassified). Unrooted, neighbor-joining phylogenetic trees were constructed with bootstrap values based on 1000 replicates.

## 3. Results

### 3.1. Construction and Characterization of the Linda Virus cDNA Clone and the Fluorophore Labeled Linda Virus Clone

**Figure 2:**
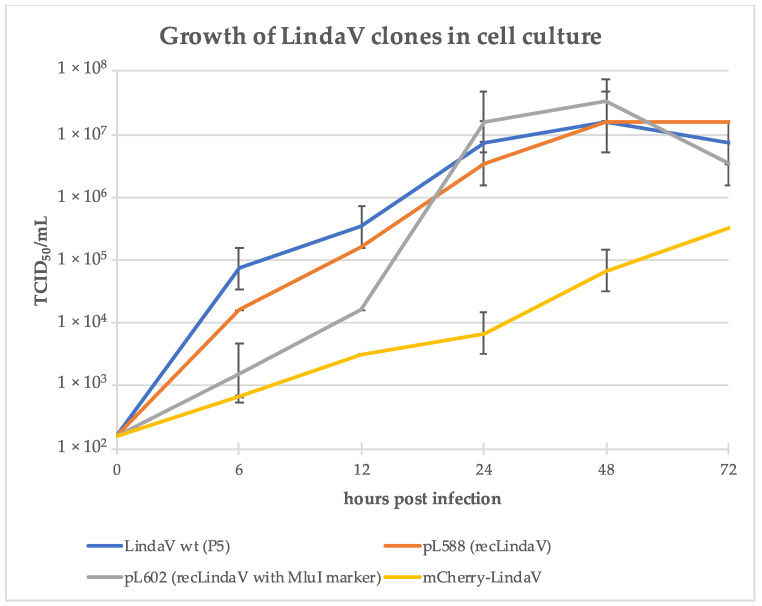
Cell culture growth of wild-type Linda virus (LindaV wt), recombinant Linda virus (recLindaV) and mCherry-Linda virus (mCherry-LindaV) clones. A monolayer of SK-6 cells was infected with 1 × 10^7^ TCID_50_ of the indicated viruses (MOI > 1). Two hours after infection, the cells were washed twice with DMEM without FCS and fresh cell culture medium was given. Cell culture supernatant samples were taken to analyze the progeny virus production after 0, 6, 12, 24, 48 and 72 h and titrated on SK-6 cells. Each titration was performed in triplicate and TCID_50_/mL was calculated using the Spearman-Kaerber algorithm. No infectious virus was found at time-point 0 h, but the limit of detection was calculated with 1.58 × 10^2^ TCID_50_/mL. Error bars represent positive and negative standard deviations.

As a first step towards a fluorescent reporter virus, a full-genome infectious cDNA clone of LindaV was generated. LindaV RNA was purified and a full-length genomic PCR product was amplified by RT-PCR. The full-length genomic PCR product was purified and cloned into a minimalistic pBR322 vector backbone from the CSFV cDNA clone p447 (as described in [29]) in line with a SP6 promoter for in vitro RNA synthesis (pL588, recLindaV). A diagnostic MluI restriction enzyme recognition site was introduced in this cDNA copy of LindaV at position nt 5587 to differentiate between wild-type and recombinant viral RNA (pL602, recLindaV with MluI marker) (results are shown in Figure S1). The replication of recLindaV in SK-6 cells was demonstrated by an indirect immunofluorescence assay using MAb 6A5 (Figure S2). Growth curves of wild-type LindaV and recLindaV with or without the genetic marker were similar, with peak titers exceeding 1 × 10^7^ TCID_50_/mL measured at 48 h post infection (Figure 2).

**Figure 3:**
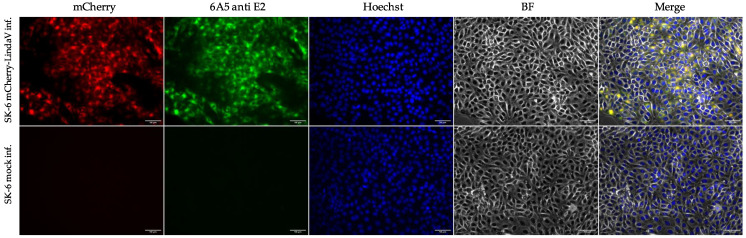
Indirect immunofluorescence assay of SK-6 cells infected with a fluorophore labeled Linda virus clone (mCherry-LindaV). SK-6 cells were infected with mCherry-LindaV and fixed at 48 h post infection. Cells were stained with the cross-reactive mouse MAb 6A5 (anti E2). Goat anti-mouse IgG conjugated with FITC was used as a secondary antibody. Cell nuclei were counterstained with Hoechst 33342. Images are shown at 20× magnification. Scale bars represent 50 µm. BF, brightfield.

Based on the LindaV cDNA clone pL588, a fluorophore labeled LindaV clone (mCherry-LindaV) was generated, where the mCherry coding sequence was inserted at the 5′-end of the LindaV E2 gene behind the signal peptide, according to recent publications on BVDV mCherry-E2 constructs [30,31]. Replication of the mCherry-LindaV in SK-6 cells was observed in infected cells showing a strong cytoplasmic fluorescence signal at 48 h post infection (first fluorescence signals were visible approximately 12 h post infection) (Figure 3). Growth of the mCherry-LindaV in SK-6 cells was reduced compared to the parental recLindaV, with titers of 3.16 × 10^5^ TCID_50_/mL at 72 h post infection (Figure 2).

### 3.2. Seroprevalence of Linda Virus in the Austrian Pig Population

The mCherry-LindaV clone was designed as a tool for the detection of LindaV specific neutralizing antibodies in porcine serum samples using an SVN assay. For validation, SVN assays with mCherry-LindaV and recLindaV were compared. Defined positive and negative reference antisera yielded the same results in both SVN assays (Figures S3 and S4), confirming the reliability of the SVN assay using mCherry-LindaV as a test virus.

**Figure 4:**
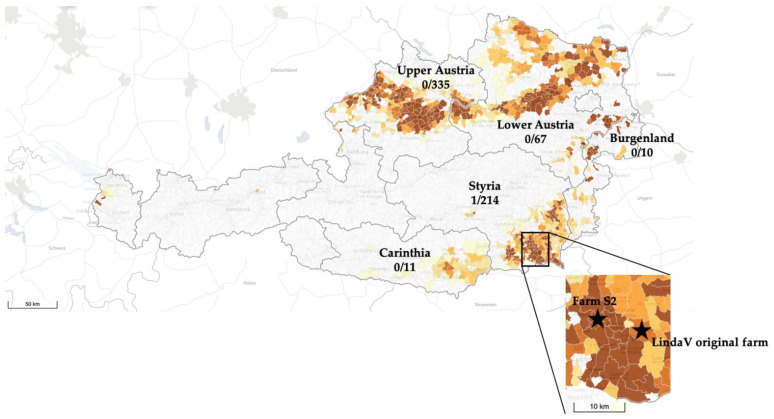
Presence of Linda virus (LindaV) neutralizing antibodies in porcine serum samples from Austrian pig farms. A total of 637 serum samples from five federal states of Austria (Upper Austria, Lower Austria, Styria, Carinthia and Burgenland) were analyzed in a LindaV SVN assay. The number of LindaV neutralizing antibody-positive sera and the total number of screened sera are given for each federal state. Colors indicate regions with high (dark brown color) and low (light brown color) pig density. The approximate locations of the antibody-positive farm (Farm S2) and the originally identified LindaV farm are marked with stars. (Modified from: https://www.statistik.at/atlas/?mapid=them_lw_as2010_viehbetriebe&layerid=layer1&sublayerid=sublayer0&languageid=0 (accessed 25 April 2021). © Statistics Austria—Cartography and GIS, created 1 September 2018).

**Figure 5:**
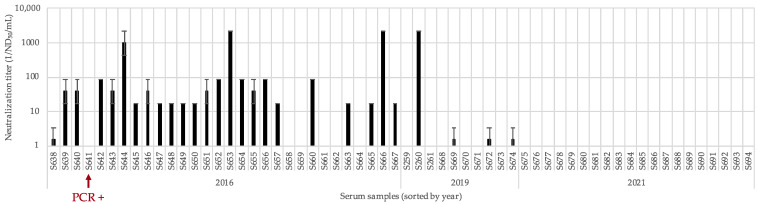
Virus neutralization titers of serum samples from Farm S2 from the years 2016, 2019 and 2021. Sera were analyzed in duplicate in a five-fold serial dilution starting at a dilution of 1/5 in an SVN assay. Neutralization titers (ND_50_/mL) were calculated using the Spearman-Kaerber method. Neutralization titers are presented as the reciprocal ND_50_ value. Error bars indicate positive and negative standard deviations. Serum sample S641 is marked with a red arrow as yielding a positive result in the RT-qPCR assay.

Sample size calculations resulted in approximately 600 porcine sera that needed to be screened to determine the seroprevalence of LindaV infections in the Austrian pig population. A total of 637 serum samples from sows and gilts, collected between the years 2015 and 2020, was analyzed. The sera originated from 132 commercial pig farms located in five federal states of Austria. As the number of pigs in the three federal states Upper Austria, Lower Austria and Styria account for approximately 93% of the whole pig population in Austria, we aimed at screening a higher number of porcine sera from these areas, depending on the availability of archived sera from sows and gilts. Taking all these aspects into consideration, we analyzed 335 sera from Upper Austria, 214 sera from Styria, 67 sera from Lower Austria, 11 sera from Carinthia and 10 sera from Burgenland. One serum from 2019, originating from a sow housed in a pig farm in Styria (Farm S2), in the distant neighborhood of the initially identified LindaV positive farm (approximately 10 km distance between the two farms), showed a neutralizing activity in the screening assay. Further analysis in a five-fold serial dilution revealed a strong neutralizing activity of 1/2180 ND_50_/mL (S2_S260, Figure 5). Two other sera from Farm S2 did not show any neutralizing activity (S2_S259 and S2_S261, Figure 5). Furthermore, no other serum of the 637 analyzed showed neutralizing activity against LindaV in the SVN assay (Figure 4).

For the detection of LindaV RNA, the 637 sera were pooled (5 sera/pool), total RNA was extracted and analyzed in a LindaV specific RT-qPCR. LindaV RNA could not be detected in any of the pooled samples. The housekeeping gene beta-actin was used as an internal control for the RT-qPCR analysis. All of the pooled sera yielded positive results in the beta-actin RT-qPCR (Ct values between 27 and 35). Three sera from Upper Austria could not be analyzed by RT-qPCR, because there was no material left for further analyses after performing the SVN assay.

The seroprevalence of LindaV was calculated to be 0.15% (1/637, based on the number of porcine sera screened) and 0.75% (1/132, based on the number of farms screened), respectively.

### 3.3. Identification of Further Linda Virus Specific Antisera in Farm S2

Fortunately, Farm S2 has been monitored repeatedly in the past because of a PRRSV circulation project and several herd health screenings. This allowed an in-depth analysis of the LindaV prevalence on this farm. Archived sera from the year 2016 (n = 30, S2_S638-S2_S667), 2019 (n = 7, S2_S668-S2_S674) and recently sent in sera from 2021 (n = 20, S2_S675-S2_S694) were analyzed by SVN assays. In the sera of post-weaning piglets and fattening pigs from 2016, LindaV neutralizing activity could be detected. While an intermediate neutralizing activity was found in 20 sera (between 1/17.2 and 1/86.4 ND_50_/mL), a high neutralizing activity between 1/968 and 1/2180 ND_50_/mL was found in three sera and a low neutralizing activity of 1/1.538 ND_50_/mL was detected in one serum, which can be considered as a negative result in the SVN assay. Three sera obtained in 2019, originating from a sow and two post-weaning piglets, showed an equally low neutralizing activity of 1/1.538 ND_50_/mL, that were also evaluated as negative results. Neutralizing activity was not detectable in any of the sera obtained in 2021 (Figure 5).

**Figure 6:**
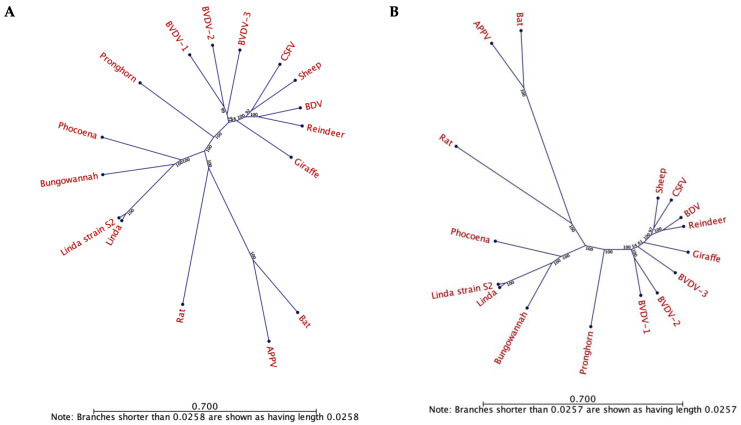
Phylogenetic analysis of the novel Linda virus strain S2 and approved and unclassified pestivirus species. Phylogenetic trees based on the full nucleotide sequence (**A**) and the polyprotein sequence (**B**) were constructed based on the neighbor-joining algorithm and bootstrap analysis with 1000 replicates and displayed as unrooted trees. Bootstrap values are indicated in percentage at each node. Scale bars indicate the number of substitutions per site. GenBank accession numbers are listed in Section 2.11. BVDV, bovine viral diarrhea virus; CSFV, classical swine fever virus; BDV, border disease virus; APPV, atypical porcine pestivirus.

All sera originating from Farm S2 were subjected to LindaV specific RT-qPCR. LindaV RNA could be detected in the serum from a post-weaning piglet from 2016 (S2_S641, Ct value 23), which did not show any neutralizing activity in the SVN assay. From the serum sample S2_S641 the full-genomic sequence of the virus was subsequently determined. A consensus sequence of 12,546 bp was established, missing only the ultimate ends of the 5′- and 3′-UTRs (GenBank accession number: MZ027894). Sequence alignment with the previously obtained LindaV sequence revealed a high identity of 98.54% based on the nucleotide sequences and of 98.37% based on the polyprotein sequences. Within the coding sequence of the envelope glycoprotein E2 we found a lower nucleotide identity of 97.87% (E2 coding sequence) and of 95.47% (E2 amino acid sequence). A phylogenetic analysis with the approved and tentative pestivirus species clustered this novel LindaV strain (LindaV strain S2) with the already described LindaV prototype from 2015, branching with the species Bungowannah virus and the recently described species Phocoena pestivirus (Figure 6).

Virus isolation by inoculation of SK-6 cells with serum sample S2_S641 was successful, despite storage at −20 °C for the last five years. Viral antigen in infected cells could be detected in immunofluorescence assays using the cross-reactive anti-pestivirus MAb 6A5 (anti E2). Successful propagation in SK-6 cells could also be demonstrated with a decrease of the Ct value in RT-qPCR, starting with a Ct value of 23 in the serum sample and resulting in a Ct value of 19 after the third passage of LindaV-S2 in SK-6 cells.

## 4. Discussion

Classical swine fever or hog cholera has been known for 200 years and the causative agent was classified in the late 1980s as a member of the newly established genus *Pestivirus* within the family *Flaviviridae* [32]. Additional porcine pestiviruses were discovered in 2003 (Bungowannah virus, *Pestivirus F*) and in 2015 (Atypical porcine pestivirus, *Pestivirus K* and Linda virus, tentatively *Pestivirus L*) [6,13,17]. While APPV proved to be globally spread in wild and domestic pig populations and sporadically causes neurodegenerative disease, BungoV and LindaV were described only locally. With regard to LindaV, there is in fact no further report from abroad [9,10] and although we routinely screen for APPV and LindaV in our diagnostic laboratory, no further detection occurred. How can it be explained that BungoV and LindaV apparently do not spread among pig populations, despite their ability to efficiently infect and replicate in the porcine host [18,33] and the ability to establish persistence after fetal infection, as has been shown for infections of the porcine fetus with BungoV [34]? Data on the outcome of an experimental infection of pregnant sows with LindaV are still missing. However, high viral loads in the sera of diseased piglets in the initially affected farm indicate a persistent infection compared to the hardly detectable viremia in experimentally infected immunocompetent pigs [18]. As a first step to elucidate the spread of LindaV, we investigated the epidemiology of this novel pestivirus in the Austrian domestic pig population by assessing the seroprevalence of LindaV specific neutralizing antibodies. The results may appear fortunate, as we identified one highly neutralizing antiserum from archived samples of a single pig herd. The resulting seroprevalence of 0.15% can only be considered preliminary due to the small sample size and requires confirmation. On the level of examined herds the suggested prevalence is 0.75%.

These numbers are in contrast to the prevalence of APPV antibodies. Studies have demonstrated a wide distribution in several countries, ranging from 9–25% seropositive animals in Germany [8] and up to ≥60% in several countries of Europe, China and Taiwan [11]. While APPV antibodies were determined by antigen detection (immunofluorescence assays or ELISAs), LindaV specific antibodies were assessed by an SVN assay that provides the maximum specificity. In a previous report we have shown that no cross neutralization with other pestivirus induced antibodies exists for LindaV, except for a BungoV antiserum [18]. Prerequisite for SVN assays is an infectious system consisting of susceptible cells and infectious virus. LindaV can be easily propagated on porcine kidney cells (SK-6) without adaptation and reaches moderate to high titers [17]. For APPV, productive infectious systems have been put forward in the last years [35], so that SVN assays can be used to confirm earlier results.

For the SVN assay we designed a reporter system using a fluorophore labeled LindaV cDNA clone. The advantage is the easy readout of the fluorescent signal of infected cells without the need of laborious indirect immunofluorescence staining procedures. Construction of the reporter virus was achieved by the fusion of an mCherry gene to the 5′-end of the LindaV E2 gene directly downstream of the E2 signal peptide coding region analogous to previously published BVDV/mCherry-E2 constructs [30,31]. The resulting mCherry-LindaV clone displayed a retardation in virus multiplication, possibly due to the size of the introduced foreign gene, yet the 10-fold lower virus titers were sufficient for the establishment of a reporter SVN assay. It is currently undetermined which antibody specifications account for the neutralizing effect, but in analogy to other pestiviruses it can be expected that E2 represents the immunodominant antigen. Neutralization assays with E2 and E^rns^ affinity purified antibodies are planned.

The screening of serum samples from Austrian pig farms revealed a second LindaV affected farm in the distant neighborhood of the initial outbreak. After the detection of a seropositive sow in our initial screening, we could identify 27 (out of 57) additional positive sera from the years 2016, 2019 and 2021 with variable neutralizing antibody titers. LindaV RNA was detectable in one serum from 2016 and virus isolation from this serum was successful. From the data available, an outbreak of LindaV in this farm is likely to have occurred in 2016, because of a large number of sera showing intermediate to high neutralizing activity at this time point and the occurrence of an antibody negative and viremic post-weaning piglet. Most of the sera from 2019 showed no neutralizing activity and none of the sera from 2021 showed any neutralizing activity. This trend could indicate that a circulation of LindaV was present in the herd for at least three years, but that the virus disappeared from the farm in later years. It is not clear, whether the viremic post-weaning piglet represents a persistently infected animal or if it was in the viremic phase of an acute infection, as there were no follow-up samples available. According to the responsible herd veterinarian and the farmer, clinical signs of congenital tremor or increased pre-weaning mortality have never been observed in this pig herd. This would suggest an infection episode with LindaV during a time where no sows were in a critical stage of gestation, a subclinical infection or a possibly less virulent LindaV strain compared to the original strain. Nevertheless, the number of samples is not representative and we only have limited information about the situation on the farm farther in the past. Surveillance of the farm assessing the serostatus and possible presence of LindaV in the pig herd is underway.

While we have not identified a direct connection between the two farms, the relatively close proximity (approximately 10 km) suggests a local transmission of LindaV. Direct transmission via transport of live, infected animals or indirect transmission, as has been shown for other pestiviruses, like BVDV, CSFV or BungoV (reviewed in [14,36,37]), can be safely assumed. LindaV excretion in nasal secretions, saliva and feces has been demonstrated as a potential route for direct or indirect horizontal transmission [18]. Another possible route of virus transmission could be the transport of slaughter pigs to the slaughterhouse, as the loading of pigs from several farms together in one trailer is a common procedure in Austria due to the small farm sizes. However, the involvement of an unknown vector or an unidentified wild reservoir host cannot be excluded. In vitro studies have demonstrated a broad cell tropism of BungoV, which is in clear contrast to other pestivirus species [38]. Cell lines of human, monkey, mouse and bat origin were susceptible, raising the question of possible reservoir hosts and the origin of this virus [38]. We are currently looking into this with LindaV, but preliminary evidence suggests a narrower host species range than BungoV, at least on the level of susceptible cell lines.

Isolation and sequencing of the new LindaV strain revealed an identity of approximately 98% to the original LindaV (KY436034.1) based on the full genomic sequence. This high sequence identity combined with nucleotide exchanges that are regularly distributed along the whole genome, could indicate a low immune selection pressure on the virus. Nevertheless, we found a slightly lower sequence identity of 97.87% within the E2 coding region and of 95.47% within the E2 amino acid sequence. These results are not surprising, as the pestiviral glycoprotein E2 is the main target for neutralizing antibodies and therefore shows the highest variability within the pestiviral genome due to immune evasion strategies.

## 5. Conclusions

With a seroprevalence of 0.15% based on the animal level, our study confirms that LindaV is a rare pathogen in Austrian domestic pigs. In future experiments we will look at the prevalence of LindaV in boars as well as in the wild boar population using SVN assays. Further epidemiological and virological studies are required to decide whether the emerging pathogen LindaV remains a rare infection or has the potential for future epidemics.

## 6. Patents

The authors B.L., L.S., and T.R. are inventors of a patent on LindaV pestivirus (PCT/EP2017/084453; Isolation of a novel pestivirus causing congenital tremor).

## Data Availability

All data analyzed or generated during this study are included in the manuscript.

## Supplementary Materials

The following are available online at https://www.mdpi.com/article/10.3390/v13061001/s1, Figure S1: Establishment of reverse genetics for Linda virus (LindaV), Figure S2: Monoclonal antibody (MAb) 6A5 detects the E2 expression in cells transfected with recombinant Linda virus (recLindaV) in an indirect immunofluorescence assay, Figure S3: Serum virus neutralization assay (SVN assay) using an mCherry-Linda virus (mCherry-LindaV) and defined positive and negative reference Linda virus antisera, Figure S4: Serum virus neutralization (SVN) assay using a recombinant Linda virus (recLindaV) and defined positive and negative reference Linda virus antisera, Table S1: Oligonucleotides used in this study.
