# Supplementary material for: Prevalence of Linda Virus Neutralizing Antibodies in the Austrian Pig Population
Source: Viruses. 2021 May 27;13(6):1001. doi: 10.3390/v13061001 (PMC8229103; doi:10.3390/v13061001)
Supplement: Supplementary file 1 [file viruses-13-01001-s001.zip › viruses-1222859 suppl 5.28.pdf]

Supplementary Material – Linda Virus Seroprevalence Paper

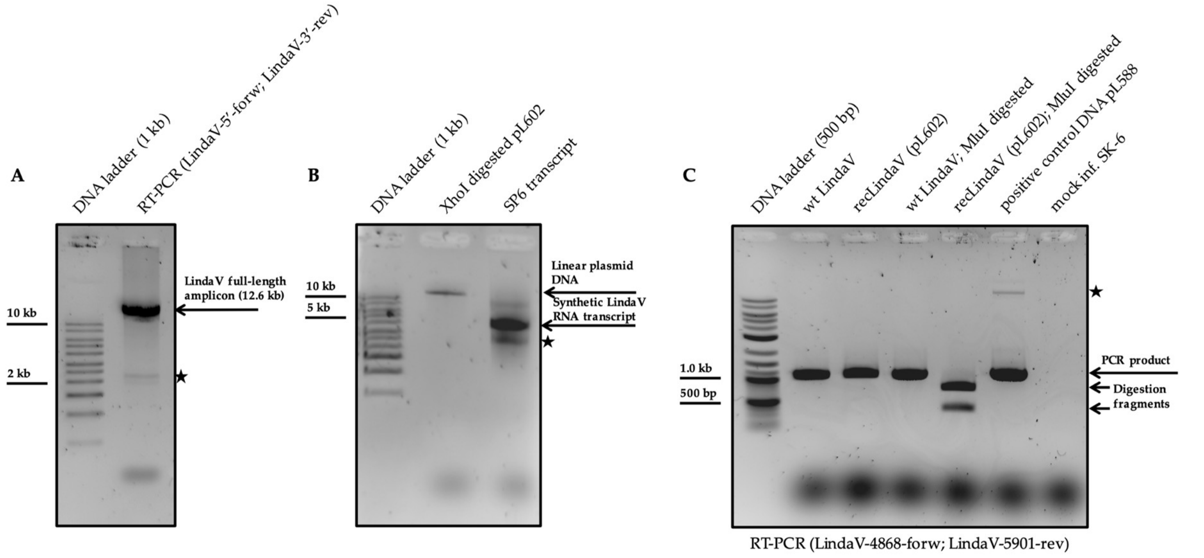

Figure S1. Establishment of reverse genetics for Linda virus (LindaV). (A) Genome length RT-PCR. Total RNA from a LindaV concentrate was transcribed using oligonucleotides hybridizing with the 5′- and 3′-ends of the LindaV genome including 5′-adapter sequences. The PCR products were subjected to agarose gel electrophoresis showing a specific LindaV genome amplicon as a strong double-stranded DNA band at 12.6 kb. Several smaller DNA bands of low intensity occur, of which two bands at about 2.0 kb labeled with asterisk. **(B)** Transcription of synthetic LindaV RNA. A molecular labeled plasmid clone of LindaV was constructed (pL602) by the integration of the LindaV DNA copy in a pBR322 backbone in between a SP6 promoter and a XhoI restriction endonuclease recognition site sequence. Plasmid DNA was digested with XhoI and transcribed using SP6 DNA dependent RNA polymerase. A strong signal of the single-stranded RNA molecule of 12.6 kb is visible after gel electrophoresis. The single-stranded RNA migrates more than two times faster than double-stranded DNA (please note the DNA marker). Weak signals of larger and smaller RNA molecules (labeled with asterisk) occur most likely due to incomplete plasmid DNA digestion and stop signal sequences for the SP6 polymerase, respectively. **(C)** Identification of the molecular marker. The infectious cDNA clone of LindaV was labeled by the integration of a synonymous MluI recognition site. A genome fragment, which includes this labeled sequence, was amplified by RT-PCR using the oligonucleotides LindaV-4868-forw and LindaV-5901-rev from cells infected with LindaV (wt LindaV) or with the molecular clone (recLindaV, pL602). After MluI digest of the PCR products, the specific DNA digestion fragments of 333 and 722 bp were only seen in cells infected with the molecular clone.

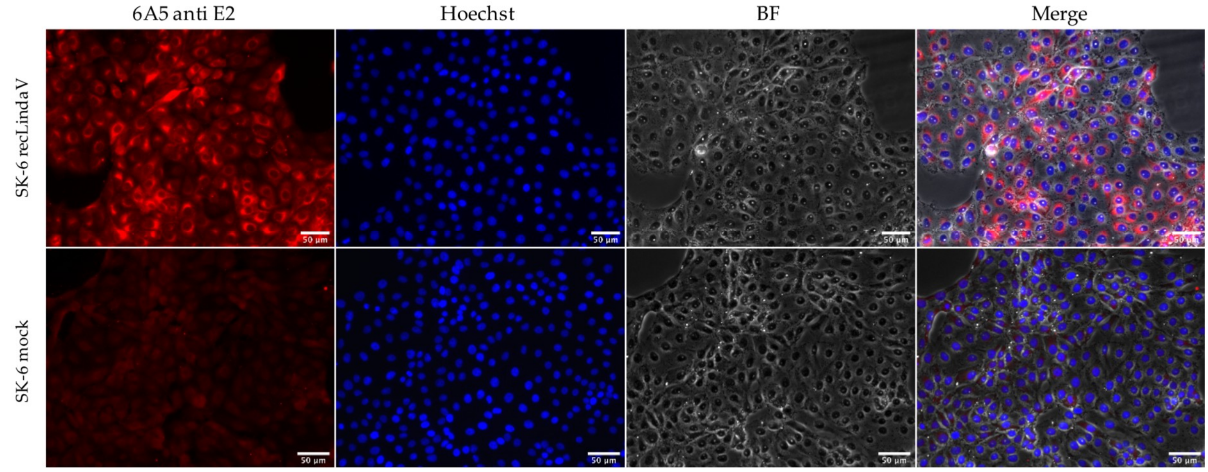

Figure S2. Monoclonal antibody (MAb) 6A5 detects the E2 expression in cells transfected with recombinant Linda virus (recLindaV) in an indirect immunofluorescence assay. SK-6 cells transfected with recLindaV were stained with the cross-reactive mouse MAb 6A5 (anti E2). Goat anti-mouse IgG conjugated with Cy3 was used as a secondary antibody. A LindaV E2 specific fluorescence is seen in cells transfected with LindaV RNA, while solely faint background signals occur in the non-transfected control cells. Cell nuclei were counterstained with Hoechst 33342. Images are shown at 20 x magnification. Scale bars represent 50 µm. BF, brightfield.

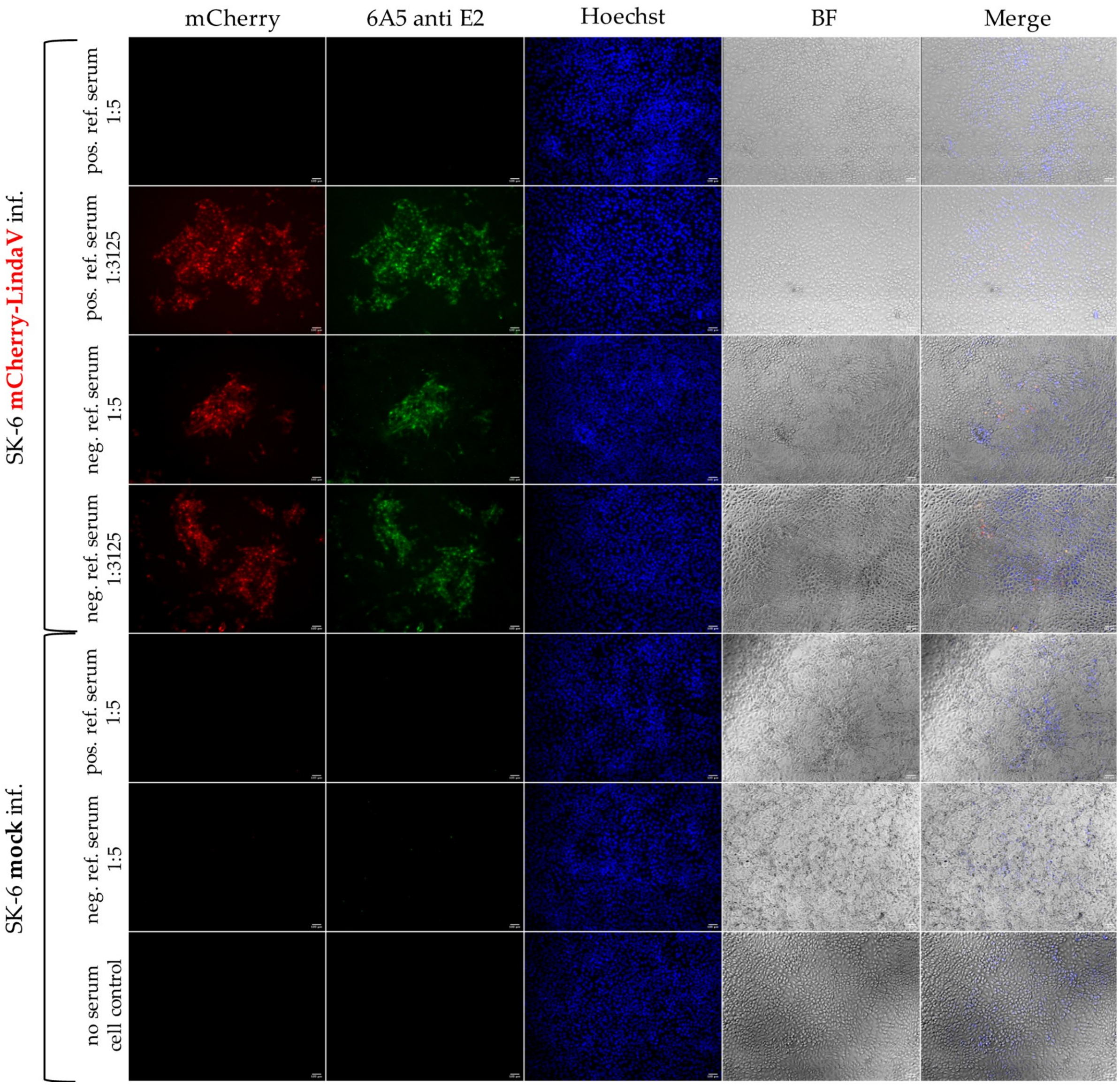

Figure S3. Serum virus neutralization assay (SVN assay) using an mCherry-Linda virus (mCherry-LindaV) and defined positive and negative reference Linda virus antisera. Five-fold serial dilutions of positive and negative antisera were prepared and incubated with an mCherry-LindaV dilution (100 $TCID_{50}$) for 2 h. SK-6 cells were added and incubated for 72-96 h. Cells were fixed and stained with the cross-reactive MAb 6A5 (anti E2). Goat anti-mouse IgG conjugated with FITC was used as a secondary antibody. Cell nuclei were counterstained with Hoechst 33342. Images are shown at 10 x magnification. Scale bars represent 100 µm. BF, brightfield.

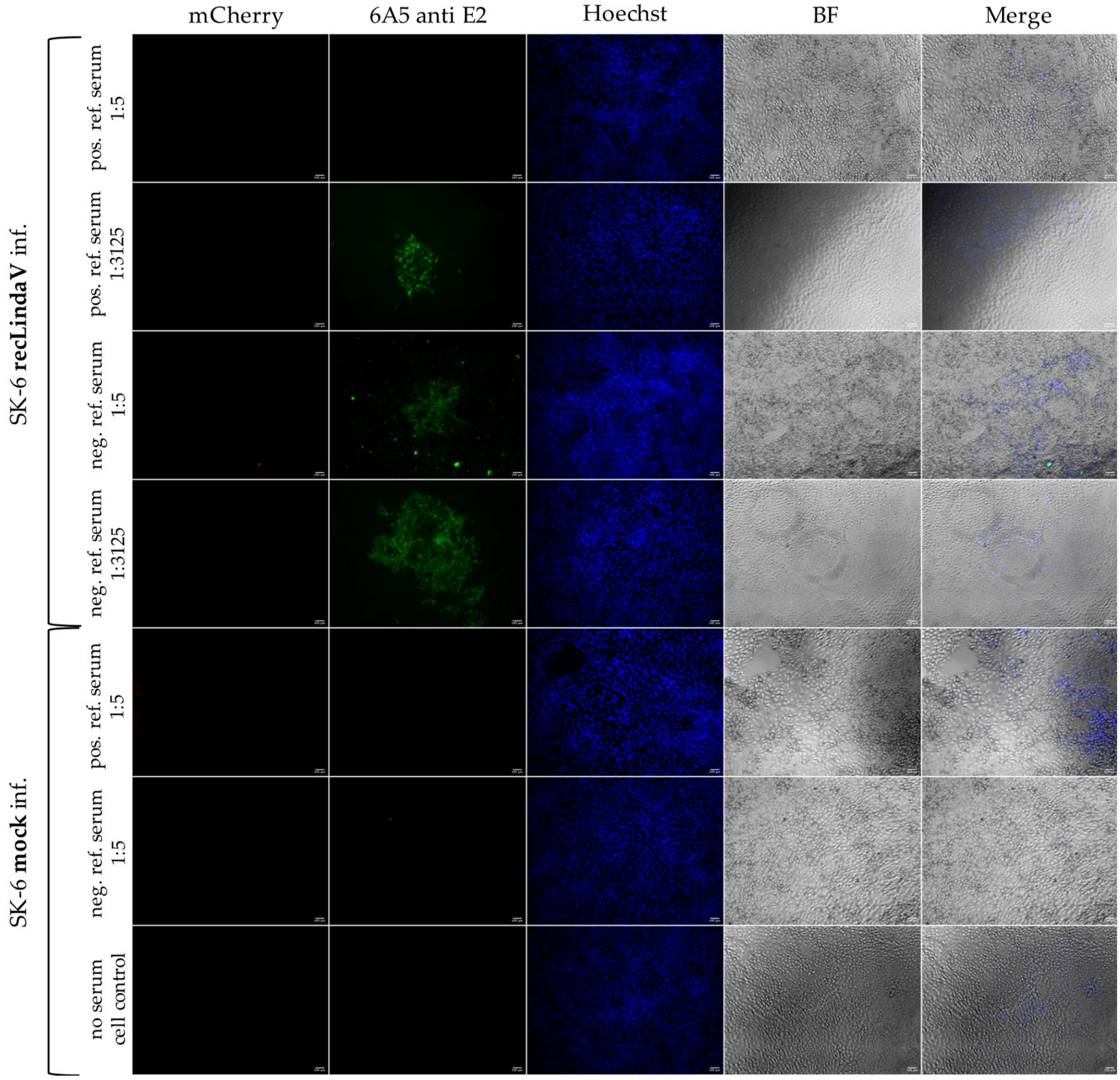

Figure S4. Serum virus neutralization (SVN) assay using a recombinant Linda virus (recLindaV) and defined positive and negative reference Linda virus antisera. Five-fold serial dilutions of positive and negative antisera were prepared and incubated with a recLindaV dilution (100 $TCID_{50}$) for 2 h. SK-6 cells were added and incubated for 72-96 h. Cells were fixed and stained with the cross-reactive MAb 6A5 (anti E2). Goat anti-mouse IgG conjugated with FITC was used as a secondary antibody. Cell nuclei were counterstained with Hoechst 33342. Note the absence of the red fluorescence signal (mCherry) compared to the images of the SVN assay with mCherry-LindaV depicted in Supplementary Figure 3. Images are shown at 10 x magnification. Scale bars represent 100 µm. BF, brightfield.

Table S1. Oligonucleotides used in this study.

Oligonucleotide name	Oligonucleotide sequence
LindaV-1-fw	5′-GTATAGCAGCAGTAGCTCAAGGCTG-3′
LindaV-544-rev	5′-GTGGTGGGTGCATGGGTCCGAAGC-3′
LindaV-446-fw	5′-ACATGTTCTGGCGGATGTACC-3′
LindaV-1496-rev	5′-TCTATATTGTACCAGTTACACCAGCC-3′
LindaV-1259-fw	5′-ACGACAAGAACGCAACAGATGTGC-3′
LindaV-2771-rev	5′-ATCAGTGTCTCAGTCGGATGGTC-3′
LindaV-2564-fw	5′-AGACTCAATGGTACCAAGCG-3′
LindaV-4024-rev	5′-ACGAAATAACTGTGAAGAGCATCGG-3′
LindaV-3796-fw	5′-ACATTGGTGTACGTAATAGGCATCG-3′
LindaV-5472-rev	5′-GCACTGCACCTTGGTTCTACCCATG-3′
LindaV-4868-fw	5′-CAGCAGACAGCAACAGTATACTATG-3′
LindaV-6821-rev	5′-CCATAACGTTGTGCCTGCAGCAG-3′
LindaV-6578-fw	5′-TGACATTACCAGACCTAGACAC-3′
LindaV-8103-rev	5′-TTCCATACCTGAGCTCACTGC-3′
LindaV-7873-fw	5′-TCGGCTCTGGCCAATTACAC-3′
LindaV-9258-rev	5′-CTCTCCAAGCTTGGTTTTGTG-3′
LindaV-9018-fw	5′-AGCACTGGTGAAGAGGTCATCC-3′
LindaV-10473-rev	5′-CTTGGACGTTTGAGCTCTGACG-3′
LindaV-10247-fw	5′-TAGAGAATCCTGGAGTGTGC-3′
LindaV-11777-rev	5′-TGTCCTATTACTTCCTTGTACGC-3′
LindaV-11559-fw	5′-GATGTGTACCAGGCTGGACTC-3′
LindaV-12607-rev	5′-GGGCCTCTTGGAACTGTAAGTAGTC-3′
